# Norepinephrine and dobutamine improve cardiac index equally by supporting opposite sides of the heart in an experimental model of chronic pulmonary hypertension

**DOI:** 10.1186/s40635-021-00391-x

**Published:** 2021-06-04

**Authors:** Janus Adler Hyldebrandt, Nikolaj Bøgh, Camilla Omann, Peter Agger

**Affiliations:** 1 Department of Anesthesia and Intensive Care, Akershus University Hospital, Postbox 1000, 1478 Lørenskog, Norway; 2 MR Research Centre, Aarhus University, Aarhus, Denmark; 3 Department of Cardiothoracic and Vascular Surgery, Aarhus University Hospital, Aarhus, Denmark; 4 Comparative Medicine Lab, Department of Clinical Medicine, Aarhus University, Aarhus, Denmark

**Keywords:** Pulmonary hypertension, Inotropes, Vasopressor, Norepinephrine, Dobutamine, Ventricular dysfunction, Experimental model, Animal model

## Abstract

**Background:**

Pulmonary hypertension is a significant risk factor in patients undergoing surgery. The combined effects of general anaesthesia and positive pressure ventilation can aggravate this condition and cause increased pulmonary blood pressures, reduced systemic blood pressures and ventricular contractility. Although perioperative use of inotropic support or vasopressors is almost mandatory for these patients, preference is disputed. In this study, we investigated the effects of norepinephrine and dobutamine and their ability to improve the arterio-ventricular relationship and haemodynamics in pigs suffering from chronic pulmonary hypertension.

**Method:**

Pulmonary hypertension was induced in five pigs by banding the pulmonary artery at 2–3 weeks of age. Six pigs served as controls. After 16 weeks of pulmonary artery banding, the animals were re-examined under general anaesthesia using biventricular conductance catheters and a pulmonary artery catheter. After baseline measurements, the animals were exposed to both norepinephrine and dobutamine infusions in incremental doses, with a stabilising period in between the infusions. The hypothesis of differences between norepinephrine and dobutamine with incremental doses was tested using repeated two-way ANOVA and Bonferroni multiple comparisons post-test.

**Results:**

At baseline, pulmonary artery-banded animals had increased right ventricular pressure (+ 39%, *p* = 0.04), lower cardiac index (− 23% *p* = 0.04), lower systolic blood pressure (− 13%, *p* = 0.02) and reduced left ventricular end-diastolic volume (− 33%, *p* = 0.02). When incremental doses of norepinephrine and dobutamine were administered, the right ventricular arterio-ventricular coupling was improved only by dobutamine (*p* < 0.05). Norepinephrine increased both left ventricular end-diastolic volume and left ventricular contractility to a greater extent (*p* < 0.05) in pulmonary artery-banded animals. While the cardiac index was improved equally by norepinephrine and dobutamine treatments in pulmonary artery-banded animals, norepinephrine had a significantly greater effect on mean arterial pressure (*p* < 0.05) and diastolic arterial pressure (*p* < 0.05).

**Conclusion:**

While norepinephrine and dobutamine improved cardiac index equally, it was obtained in different manners. Dobutamine significantly improved the right ventricular function and the arterio-ventricular coupling. Norepinephrine increased systemic resistance, thereby improving arterial pressures and left ventricular systolic function by maintaining left ventricular end-diastolic volume.

## Background

Patients with pulmonary hypertension have a sevenfold increased 1-year standardised mortality ratio and are hospitalised with greater frequency [1]. These patients carry an elevated risk of perioperative mortality (1–18%) [2–5, 7], and morbidity (14%–42%) [2–6]. In the perioperative setting, they are more likely to develop heart failure, sepsis, respiratory failure, spend longer time on ventilator support and in the ICU [2–6].

With disease progression, the increased pressure in the right ventricle will compress the left ventricle by leftwards deviation of the interventricular septum, hence decreasing left ventricular filling and compromising diastolic [8, 9] and systolic function [10]. Positive pressure ventilation, systemic hypotension, acidosis, hypoxia, hypercapnia and tachycardia will all aggravate pulmonary hypertension. Hence, administering general anaesthesia and providing perioperative care for major surgeries in this patient group can be a daunting task.

The choice of perioperative inotropic support or vasopressors for these patients is disputed and relies on limited data. Norepinephrine, dopamine, adrenaline, dobutamine, levosimendan and milrinone have all been suggested and used [9, 11, 12]. Most animal studies use acute models with short periods of pulmonary artery constriction when analysing the effect of inotropic therapy in the failing pressure-overloaded right ventricle [11–13]. However, taking into account the multitude of anatomical [14, 15], histological [16] and biochemical changes [17–19] in chronic RV failure, lessons from acute studies should not be viewed as definite proofs in regard to chronic RV failure conditions.

The ideal treatment for the stabilisation of the chronically pressure-overloaded failing right ventricle in the perioperative environment involves increasing systemic pressure, improving myocardial perfusion, increasing biventricular myocardial contractility and stroke volumes while avoiding increases in pulmonary artery resistance. In the failing right ventricle, the treatment effect is summarised by the ratio of pulmonary artery elastance and the right ventricular contractility also known as the arterio-ventricular relationship of the failing right ventricle.

With this in mind, we undertook an experimental animal study of pressure-overloaded right ventricular failure and studied the effects of norepinephrine and dobutamine, as they have distinctly different haemodynamic effects. Norepinephrine is a potent α_1_-adrenergic receptor agonist with low β-agonist activity, and therefore a powerful vasoconstrictor. It has only direct inotropic properties at high doses. Dobutamine is a synthetic catecholamine with a strong affinity for both β_1_- and β_2_-receptors and low α_1_-adrenergic agonism. The combined effect is most often mild vasodilation at lower doses and strong inotropic effect at higher doses.

We chose a model of moderate pulmonary hypertension, as this group is much more likely to undergo general anaesthesia and pressure overload ventilation when compared to patients with severe pulmonary arterial hypertension, in which case regional anaesthesia would be preferred. Clinically relevant dosages of norepinephrine and dobutamine were used, and haemodynamics and arterio-ventricular coupling, measured by the conductance catheter technique were chosen as the main endpoints.

## Materials and methods

This study was conducted in conformance to the Guide for the Care and Use of Laboratory Animals published by the US National Institutes of Health [20] and the ARRIVE Guidelines [21] and was approved by the Danish Animal Experiments Inspectorate (License number: 2015-15-0201-00744).

Danish landrace piglets aged 2–3 weeks (≈ 8 kg) underwent surgical pulmonary artery banding (*n* = 5). The pulmonary artery was exposed through a left lateral thoracotomy. Growth-induced stenosis was created by fitting two silicone bands around the pulmonary artery, as described in a previous study [18]. After a growth period of 16 weeks, the animals developed right ventricular dysfunction and were re-examined while under anaesthesia and compared with an age- and weight-matched control group (*n* = 6).

### Pulmonary artery banding

The animals were pre-medicated immediately upon arrival at the surgical facilities with midazolam (0.5 mg/kg) and azaperone (0.5 mg/kg), to allow for the establishment of intravenous access. Propofol (3 mg/kg) and pancuronium (0.1 mg/kg) were administered to allow endotracheal intubation. The animals were ventilated (S/5 Avance Datex Ohmeda, GE Healthcare, WI, USA) with a tidal volume of approximately 10 mL/kg, a fraction of inspiratory oxygen of 35% and a positive end-expiratory pressure of 4 cm H_2_O. Ventilation was adjusted to maintain partial pressure of carbon dioxide in the arterial blood at approximately 5 kPa. Arterial blood gases were analysed, and lactate concentration measured (ABL800; Radiometer) every 30 min to ensure normal ventilation and oxygenation. Lactate levels were also analysed. Body temperature was maintained within the normal range (38–39 °C) using a warming blanket. Continuous analgesia and anaesthesia were maintained with Fentanyl (25 μg/kg/h) and Sevoflurane inhalation at 2.7%. Prior to surgery, the animals were administered prophylactic antibiotics (penicillin 20,000 IU/kg (Ceva Animal Health, Vejle, Denmark) and analgesics (Flunixin 25 mg), both as intramuscular injections. Following the surgery, the animals were aroused and extubated when they started moving and breathing steadily on their own.

Over the following 5 days, veterinary technicians administered the animals with Flunixin 25 mg and penicillin (100.000 IU) once daily and paracetamol 250 mg (Actavis, Gentofte, Denmark) orally twice daily.

### Re-examination

On the day of re-examination, the animals were anaesthetised and ventilated using the same protocol but omitting pancuronium. Four sheaths were inserted bilaterally into the jugular veins and carotid arteries of the animals. They were then administered 150 IU/kg of heparin prior to catheter insertion, to avoid clotting within the sheaths. Following the administration of 500 μg fentanyl euthanasia was performed by excision of the heart.

### Haemodynamic measurements

A Swan-Ganz catheter (7.5F CCOmbo, Edwards Lifescience, Irvine, USA) was inserted into the pulmonary artery to continuously determine cardiac output (CO) and mixed venous oxygen saturation (SvO_2_, %). Mean arterial pressure (MAP, mmHg), mean pulmonary artery pressure (mPAP) and central venous pressure (CVP, mmHg) were measured at the level of the phlebostatic axis and recorded continuously using pressure transducers (TruWave, Edwards Lifescience, Germany) throughout the experiment. Heart rate (HR, beats/min) was determined from the arterial pressure curve.

### Ventricular measurements

Volumes were calibrated using cine MRI images obtained prior to catherisation [18]. Guided by fluoroscopy, pressure–volume catheters (Ventric-Cath 510, Millar Instruments, USA) were inserted antegrade into the right ventricle and retrograde into the left ventricle. A Fogarty occlusion catheter (Boston Scientific, Denmark) was positioned in the inferior caval vein to induce preload reduction. Calibration and preload occlusion measurements were performed for each ventricle, with the other catheter disconnected to avoid the possibility of crosstalk.

The maximum rate of pressure change (d*P*/d*t*_max_, mmHg s^−1^), preload recruitable stroke work (PRSW, mmHg ml ml^−1^), end-systolic pressure–volume relationship (ESPVR, mmHg ml), end-systolic pressure–volume relationship *x*-axis intercept (ESPVR *V*_o_, ml) and maximum ventricular pressure (*P*_max,_ mmHg) were used as measures of systolic function. The end-diastolic pressure–volume relationship (EDPVR, mmHg ml^−1^) was used as a parameter for ventricular stiffness, and the isovolumic relaxation constant (Weiss method) (tau, ms) was employed as a parameter for active diastolic relaxation. Arterial elastance (Ea mmHg ml^−1^), a measure of the arterial load, was calculated as the ventricular end-systolic pressure (mmHg) divided by the stroke volume (ml) and calculated for both ventricles. The arterio-ventricular coupling was defined as ESPVR divided by Ea. Signals were sampled using an MPVS Ultra (Millar Instruments, USA)*,* processed in PowerLab 16/35 (ADinstruments, UK), recorded at 2 kHz and analysed in LabChart 7 Pro (Adinstruments, UK).

### Calculations

The systemic vascular resistance index (SVRI, dyn·s/cm^5^/kg) and the pulmonary vascular resistance index (PVRI, dyn·s/cm^5^/kg) were calculated as 80 (MAP – CVP)/CO/kg and 80 (right ventricular mean pressure – left ventricular minimum pressure (*P*_min_))/CO/kg, respectively. CO, stroke volume (SV) and ventricular volumes were indexed to body surface area (BSA, m^2^) [21].

### NT-proBNP

Venous blood was sampled at 16 weeks, following pulmonary artery banding. Lithium–heparin-coated vacutainers were used for sampling and centrifuged for 15 min at 1000×*g* at 2–8 °C. The plasma was stored at − 80** °C**. All samples were thawed and analysed at the same time. NT-proBNP was assessed using an ELISA KIT (Porcine N-terminal pro-brain natriuretic peptide ELISA Kit (Porcine NT-proBNP, Mybiosource, USA).

### Medication protocol

**Fig. 1:**
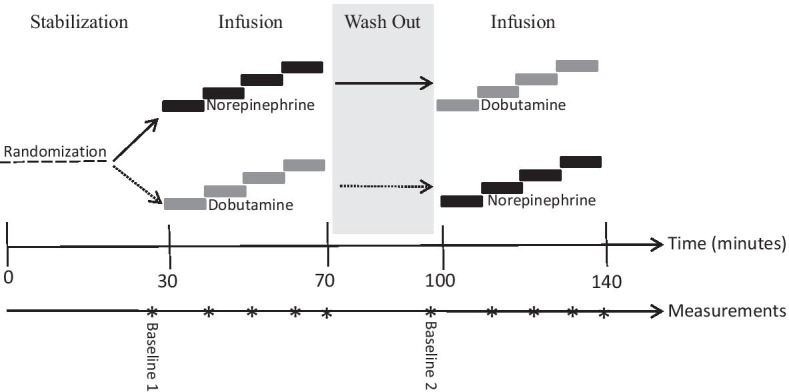
Study design: Crossover design with wash out period. Animals were randomised to start treatment with either norepinephrine or dobutamine in incremental doses. After a wash out period the animals were then treated with the other drug. *Measurement

Following instrumentation, the animals were subjected to a 30-min stabilisation period, after which baseline measurements were taken. The animals were then randomised to start incremental intravenous infusions with either norepinephrine (0.05, 0.1, 0.25, 0.5 µg kg min^−1^) or dobutamine (1.0, 2.5, 5.0, 10 µg kg min^−1^). Each step was performed for 10 min and concluded with new measurements. After a washout period of 30 min, new baseline measurements were taken and the second treatment was started in incremental steps (Fig. 1). Concentrations were selected after pilot experiments (*n* = 3, not included in data) were conducted to achieve approximately 100% increase in contractility, estimated by PRSW for both norepinephrine and dobutamine.

### Statistics

Differences between the baseline measurements of pulmonary artery-banded animals and controls were measured at the first baseline and presented as mean (± SD). Significant differences were assessed using a two-tailed Student’s *t*-test for normally distributed data and Wilcoxon rank-sum test for non-parametric data. Normal distribution was verified using quantile–quantile plots and histograms.

The hypothesis of differences between norepinephrine and dobutamine with incremental doses was tested using repeated two-way ANOVA (between-group differences) and Bonferroni multiple comparisons post-test, using the GraphPad Prism 5.04 software (GraphPad Software Inc., La Jolla, California, USA). If significant difference was detected between baseline and post-washout, the data were analysed as relative changes from baseline. A *p*-value of 0.05 was considered to be statistically significant.

## Results

### Effect of pulmonary artery banding

No differences were found between the two groups in terms of animal age, weight and BSA (Table 1). CI was significantly lower in PAB animals (− 23%, *p* = 0.04) due to lower SV (− 11%, *p* < 0.05). SVRI and left ventricle (LV) Ea were equal between PAB animals and controls. However, a significantly low SAP (− 13%, 0 = 0.02) was observed in PAB animals. Pulmonary resistance was significantly higher in the PAB animals when compared to the controls, as assessed with both PVRI (+ 156, *p* = 0.005) and right ventricular (RV) Ea (+ 135%, *p* = 0.001).

**Table 1:** Haemodynamics, ventricular function and volumes and heart failure markers in pulmonary artery-banded (PAB) animals and controls

	Control (*n* = 6)	PAB (*n* = 5)	*p*
Biometrics			
Body weight (kg)	37.7 (0.9)	37.8 (4.5)	1.0
BSA (m^2^)	0.8 (0.01)	0.8 (0.06)	1.0
Haemodynamics			
HR (min^−1^)	81.8 (11.3)	71.2 (7,9)	0.1
CI (L/min/m^2^)	5.3 (1.1)	4.1 (0.5)	**0.04**
SVi (ml/m^2^)	64.6 (6.8)	57.5 (2.2)	** < 0.05**
SAP (mmHg)	90.2 (1.7)	78 (6)	**0.01**
DAP (mmHg)	58 (9)	52 (7)	0.2
MAP (mmHg)	71.8 (9)	63.8 (7.8)	0.2
SVRI (dyn·s/cm^5^/kg)	1226 (355)	1423 (97)	0.2
PVRI (dyn·s/cm^5^/kg)	140 (81)	358 (99)	**0.005**
RV Ea (mmHg/ml)	0.58 (0.09)	1.36 (0.4)	**0.001**
LV Ea (mmHg/ml)	1.76 (0.28)	2.09 (0.82)	0.4
Ventricle			
RV *P*_max_ (mmHg)	27.4 (1.9)	44.7 (9.7)	**0.02**
LV *P*_max_ (mmHg)	84 (5.6)	68 (4.8)	** < 0.05**
RV *P*_mean_ (mmHg)	15.1 (1.0)	23.2 (5.1)	**0.02**
LV *P*_mean_ (mmHg)	39.9 (5.0)	42.7 (5.3)	0.4
RV *P*_min_ (mmHg)	7.1 (1.1)	6.6 (3.1)	0.7
LV *P*_min_ (mmHg)	7.6 (1.8)	7.6 (1.4)	1.0
RV ESV (ml)	36.6 (6.9)	32.7 (18)	0.7
LV ESV (ml)	40.2 (6.7)	12.7 (8.1)	**0.03**
RV EDV (ml)	65.3 (19.8)	77.2 (10.1)	0.2
LV EDV (ml)	82.3 (7.9)	55 (4.9)	**0.02**
RV PRSW (mmHg·ml·ml^−1^)	10 (3.5)	15.2 (5.2)	0.08
LV PRSW (mmHg·ml·ml^−1^)	44 (8.8)	41.8 (17.9)	0.8
RV ESPVR (mmHg·ml^−1^)	0.35 (0.12)	1.07 (0.4)	**0.02**
LV ESPVR (mmHg·ml^−1^)	0.9 (0.08)	1.32 (0.5)	0.5
RV ESPVR *V*_0_ (ml)	− 31.8 (30.4)	2.4 (11.7)	**0.04**
LV ESPVR *V*_0_ (ml)	− 38.3 (16.9)	− 42.6 (18.3)	0.7
RV d*P*/d*t*_max_ (mmHg/s)	387.2 (93.6)	542.8 (52.7)	**0.01**
LV d*P*/d*t*_max_ (mmHg/s)	1081 (240)	1385 (322)	0.1
RV Ea/ESPVR	1.88 (0.8)	1.34 (0.3)	0.1
LV Ea/ESPVR	1.78 (0.43)	1.8 (0.57)	0.96
RV EF (%)	52.6 (6.6)	52.8 (14)	1.0
LV EF (%)	56 (5)	71 (12)	**0.03**
RV d*P*/d*t*_min_ (mmHg/s)	− 378.5 (95)	− 677.5 (164.5)	**0.004**
LV d*P*/d*t*_min_ (mmHg/s)	− 1680 (121)	− 1395 (324)	0.1
RV tau (ms)	75.4 (13.8)	76.8 (17)	0.9
LV tau (ms)	47 (5.3)	62 (13.4)	**0.03**
RV EDPVR (mmHg/ml)	0.16 (0.04)	0.15 (0.05)	0.7
LV EDPVR (mmHg/ml)	0.18 (0.06)	0.23 (0.05)	0.4
Biochemistry			
Lactate (mM)	1.0 (0.3)	1.7 (0.3)	**0.007**
NT-pro-BNP (pg/ml)	6.3 (7.0)	19.3 (8.9)	**0.02**

Data are presented as means (SD)

BSA, body surface area. CI, cardiac index. DAP, diastolic artery pressure. dP/dt_max_, maximum pressure development over time. dP/dt_min_, minimum pressure development over time. Ea: arterial elastance. EDV, end-diastolic pressure. EDP, end-diastolic pressure. EDPVR, end-diastolic pressure–volume relationship. EF, ejection fraction. ESPVR, end-systolic pressure–volume relationship. ESPVR V_0_, end-systolic pressure–volume relationship x-axis intercept. ESP, end-systolic pressure. ESV: end-systolic pressure. HR, heart rate. LV, left ventricle. MAP, mean artery pressure. NT-proBNP: N-terminal pro-brain natriuretic peptide. P_ed_, minimum ventricular pressure. P_mean,_ mean ventricular pressure. P_max_, maximum ventricular pressure. PRSW, preload recruitable stroke work. P_ed_, minimum ventricular pressure. PRSW, preload recruitable stroke work. RV, right ventricle. PVRI, pulmonary vascular resistance index. SAP, systolic artery pressure. SVi: stroke volume index. SVRI, systemic vascular resistance index. tau, isovolumic relaxation constant

In the PAB animals, the RV had significantly increased ejective force, as shown by greater RV *P*_max_ (+ 63%, *p* = 0.02), RV *P*_mean_ (+ 56%, *p* = 0.02), RV ESPVR (+ 205, *p* = 0.02), and RV d*P*/d*t*_max_ (+ 40%, *p* = 0.01) (Table 1). RV PRSW differences did not meet a level of significant difference (*p* = 0.1). The atrioventricular relationship was not significantly changed (*p* = 0.1) and RV EF was maintained (*p* = 1.0). Diastolic properties were unchanged in both active (RV Tau *p* = 0.9) and passive ventricular relaxation (RV EDPVR, *p* = 0.4).

In the LV, *P*_max_ (− 19%, *p* < 0.05), EDV (− 33%, *p* = 0.02) and ESV (− 68%, *p* = 0.03) were lower and LV EF (+ 27%, *p* = 0.03) was significantly higher in the PAB animals when compared to the controls. LV PRSW, LV ESPVR and LV d*P*/d*t*_max_ were unchanged between the two groups (*p* > 0.05). Of the diastolic parameters, only Tau was significantly different between the two groups.

NT-proBNP concentrations were significantly higher in the PAB animals when compared to the controls, as were arterial lactate levels (Table 1).

### Effects of norepinephrine and dobutamine

**Fig. 2:**
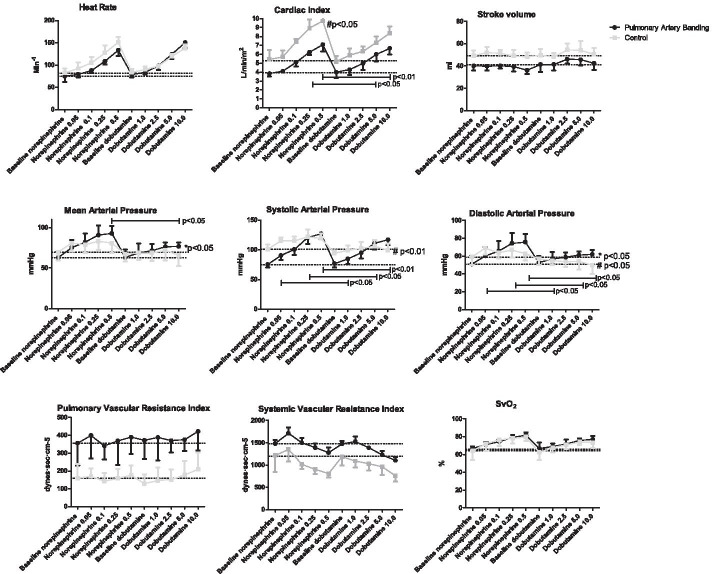
Haemodynamics in pulmonary artery-banded and control animals subjected to incremental norepinephrine and dobutamine infusions. #Significant difference between the effect of norepinephrine and dobutamine infusions in control animals. *Significant difference between the effect of norepinephrine and dobutamine infusions in pulmonary artery-banded animals. Bars and associated *p*-values indicate differences at specific time points in groups were a significant between-group differences was detected (Bonferroni). Grey square: control. Black circle: pulmonary artery banding. Dotted lines indicate baseline 1 level. Data presented as mean (SD). SvO_2_: mixed venous oxygen saturation

Differences in heart rate and stroke volume between treatment groups in both control and PAB animals did not reach statistical significance (*p* > 0.05). However, a significantly greater CI was observed in norepinephrine-treated controls, at the highest infusion concentrations (*p* < 0.05 and *p* < 0.01), respectively (Fig. 2).

Norepinephrine had a significantly greater effect on MAP (+ 18% at highest infusion rate, *p* < 0.05) and DAP (+ 18%, *p* < 0.05) when compared to dobutamine in PAB animals, but had equal effects on SAP (*p* > 0.05). In controls, norepinephrine increased SAP (*p* < 0.01) and DAP (*p* < 0.05) significantly more than dobutamine, but no difference was found in MAP (*p* > 0.05) (Fig. 2). Dobutamine did not alter SAP, DAP or MAP significantly in the control animals (*p* < 0.05). SVRI decreased in both groups with increasing doses (*p* < 0.0001), but no differences were found between the treatment groups in both PAB and control animals (*p* > 0.05). PVRI did not change with increasing doses of norepinephrine or dobutamine in both PAB and control animals (*p* > 0.05) (Fig. 2).

**Fig. 3:**
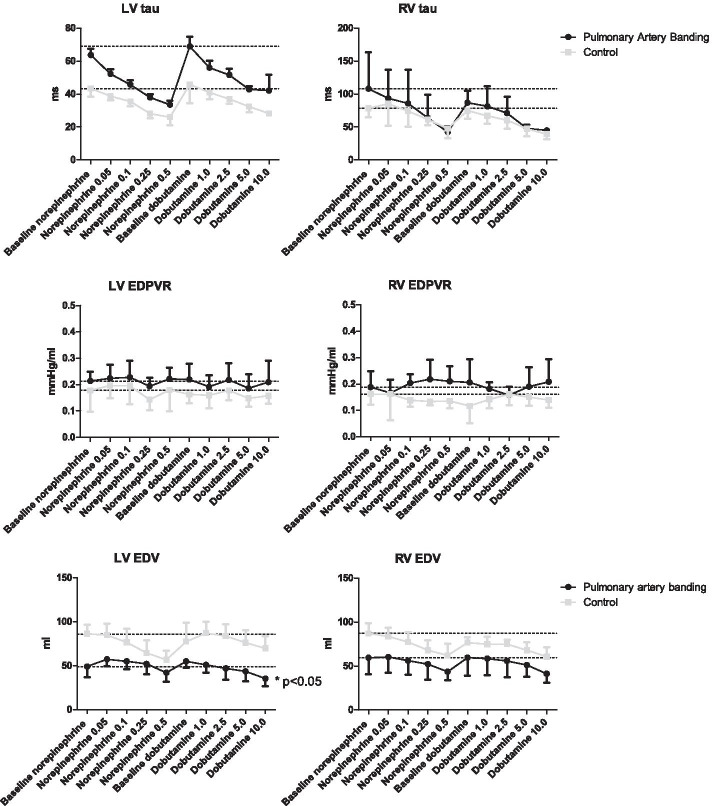
Diastolic variables in pulmonary artery-banded and control animals subjected to incremental norepinephrine and dobutamine infusions. LV: left ventricle. RV: right ventricle. EDPVR: end-diastolic pressure volume relationship. Grey square: control. Black circle: pulmonary artery banding. Dotted lines indicate baseline 1 level. Data presented as mean (SD)

**Fig. 4:**
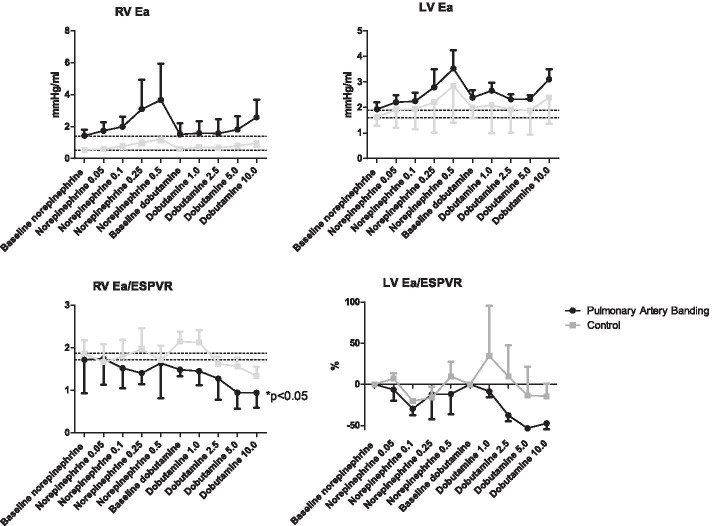
Arterio-ventricular coupling in pulmonary artery-banded and control animals subjected to incremental norepinephrine and dobutamine infusions. LV: left ventricle. RV: right ventricle. Ea: arterial elastance. ESPVR: end-systolic pressure volume relationship. *Significant difference between the effect of norepinephrine and dobutamine infusions in pulmonary artery-banded animals. Grey square: control. Black circle: pulmonary artery banding. Dotted lines indicate baseline 1 level. Data presented as mean (SD)

**Fig. 5:**
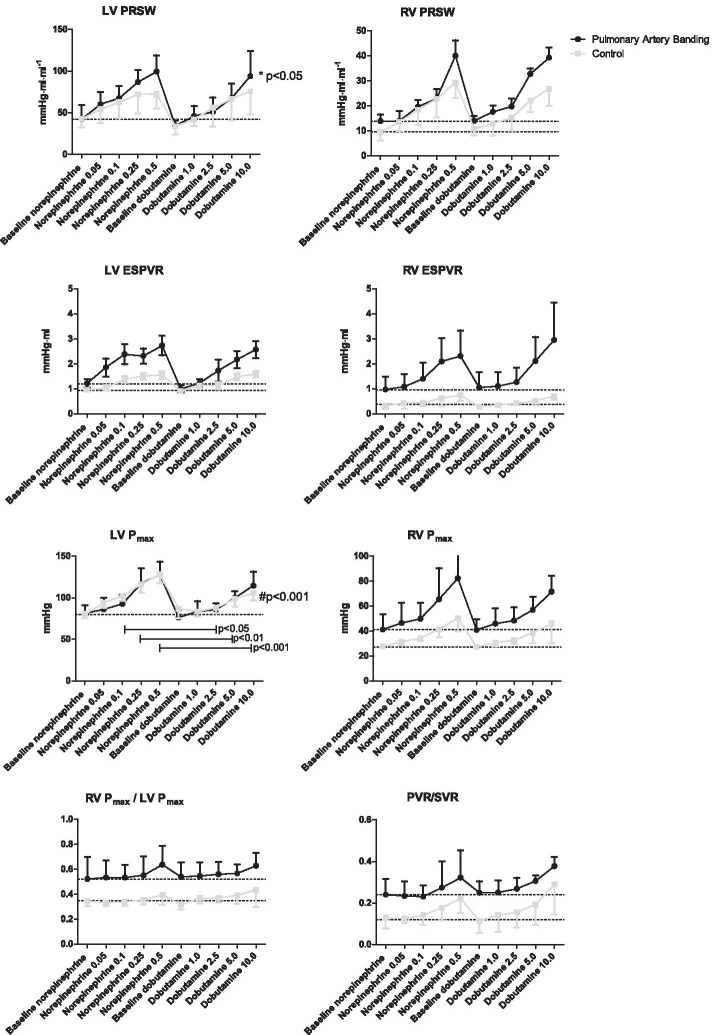
Ventricular variables in pulmonary artery-banded and control animals subjected to incremental norepinephrine and dobutamine infusions. LV: left ventricle. RV: right ventricle. PRSW: preload recruitable stroke work. ESPVR: end-systolic pressure volume relationship. Ea: arterial elastance. *P*_max_: maximum ventricular pressure. ^#^Significant difference between the effect of norepinephrine and dobutamine infusions in control. *Significant difference between the effect of norepinephrine and dobutamine infusions in pulmonary artery-banded animals. Bars and associated *p*-values indicate differences at specific time points in groups were a significant between-group differences was detected. Grey square: control. Black circle: pulmonary artery banding. Dotted lines indicate baseline 1 level. Data presented as mean (SD)

Left ventricular contractility was increased to a greater extent by norepinephrine than dobutamine in the PAB animals as judged by PRSW (*p* < 0.05) and *P*_max_ (*p* < 0.001), but no difference was observed in ESPVR. No difference in LV contractility was observed in controls. LV EDV decreased with increasing doses in both treatment groups. However, LV EDV was significantly higher during norepinephrine treatment compared to dobutamine (*p* < 0.05) (Fig. 3). Norepinephrine and dobutamine had equal inotropic effects (PRSW, ESPVR both *p* > 0.05) in the right ventricle and increased RV systolic pressures (Pmax, *p* > 0.05) to the same level. RV Ea/ESPVR was improved significantly more by dobutamine (− 36%, *p* < 0.05) in PAB animals compared to Norepinephrine (− 4%), while there were no differences in LV Ea/ESPVR (Fig. 4). The relationship between maximum pressures in the ventricles (LV *P*_max_/RV *P*_max_) did not change with increasing infusion rates in any of the groups (*p* < 0.05) (Fig. 5).

Tau, the measure of active relaxation, was significantly decreased in both groups in both ventricles with increasing infusion rates, but no differences were found between the treatment groups. Passive relaxation in the form of EDPVR remained unchanged in both groups at all infusion rates. EDV was reduced significantly with increasing doses in both ventricles (*p* < 0.05), but without any differences between the groups (Fig. 3).

## Discussion

Following 16 weeks of pulmonary artery banding, right ventricular dysfunction had developed with an increased right ventricular pressure and septal left shift, while there was concomitant decrease in left ventricular volumes and pressures. These changes were associated with haemodynamic deterioration and reduced diastolic function. Increased NT-proBNP and arterial lactate concentrations indicated biochemical hallmarks of cardiac dysfunction when compared with control animals. We have previously presented data using the same model, showing how myocardial metabolism has shifted towards anaerobic metabolism after 16 weeks, which is a hallmark of heart failure, at the time of examination [18].

This is the first study to present data on the effect of norepinephrine and dobutamine in a model of chronic right ventricular pressure overload. Norepinephrine and dobutamine had equal effects on cardiac index in pulmonary artery-banded animals. Norepinephrine produced greater left ventricular contractility combined with higher systemic pressures, while dobutamine improved the arterio-ventricular coupling of the right ventricle.

The optimal circulatory perioperative management of patients with pulmonary hypertension is focused on increasing or maintaining the mPAP and PVRI/SVRI ratio, thereby normalising the interventricular septum displacement and improving the arterio-ventricular coupling of the right ventricle. Research data conforming to large animal models with chronic pulmonary artery hypertension on the topic of inotropic support is scarce. In an experimental model of acute right ventricular pressure overload failure, Kerbaul et al. [11] found that dobutamine increased cardiac output to a greater extent than norepinephrine, due to improvement in right ventricular contractility and arterio-ventricular coupling at doses comparable to the present study. In the present study too, dobutamine was found to be more efficient in improving the right ventricular arterio-ventricular coupling. On the other hand, norepinephrine produced greater left ventricle systolic function (PRSW and *P*_max_). Also, the end-diastolic volume was found to be significantly higher during norepinephrine treatment. Logical conclusions can be drawn to justify the differences between the results of the two studies. In chronic pressure overload of the right ventricle, gradual muscular development was induced to compensate for the increase of right ventricle afterload. With right ventricle systolic function relatively well-preserved, the relative effect of dobutamine will be minor and therefore not remedy the effect of the reduced left ventricle end-diastolic volume.

Though systemic vascular resistance index was not statistically greater in the norepinephrine group, the greater afterload was enough to improve left ventricle ejection force by normalising the geometry of the left ventricle and improving systolic and diastolic function in accordance with the literature [10, 13, 22].

At the baseline, systemic pressures were significantly lower in the banded animals. In the perioperative setting, a treatment to counterweight the hypotensive effect of general anaesthesia will be required, and as such dobutamine was found to be ineffective. This point is especially important for centres that use inodilators such as milrinone and levosimendan in combination with a pressor/inotrope during stabilisation of these patients [23].

Diastolic active relaxation was significantly reduced in banded animals, in accordance with general literature and our own previous findings [8, 24]. This is commonly understood as a consequence of interseptal bowing to the left, from a pressure and volume overloaded right ventricle [8]. An important finding of this study was the relatively greater improvement noted during treatment in the pulmonary artery-banded animals, when compared with controls. Improved active relaxation (Tau) was found to be at an even degree in both norepinephrine and dobutamine treatments, which is a positive improvement in cardiac function, previously overlooked in studies on inotropic support for this patient group.

Passive relaxation (EDPVR) did not differ between controls and banded animals and was unaffected when the doses of dobutamine and norepinephrine were increased. The preserved passive relaxation points to an unaltered extracellular matrix and no fibrosis. This would be consistent with previous studies, wherein no or little fibrosis was found [16, 25, 26] in pigs having volume overload right ventricular dysfunction.

This study underlines the importance of a thorough pre- and perioperative assessment in this patient group. In patients with pulmonary hypertension and reduced left ventricular end-diastolic volume, deterioration of haemodynamics must be expected during induction of anaesthesia and positive pressure ventilation. Albeit increasing heart rate and thereby reducing filling time, norepinephrine kept the left ventricular end-diastolic volume constant, essentially at net increase. In a previous study that used the same model as the present study [9], we found significant decreases in left ventricular end-diastolic volume and arterial pressures, with increasing doses of both epinephrine and dopamine. These results, in combination with those obtained from the present study; suggest that norepinephrine is a superior first line vasopressor/inotrope.

### Limitations

Although this study has used a model of pulmonary artery constriction over a timespan longer than that used in any previous study, the degree of resemblance to patients with pulmonary hypertension is uncertain. We did not investigate myocardial fibrosis or intracellular changes as is observed in advanced heart failure [27].

## Conclusion

When exposing pigs to chronic pressure overload pulmonary hypertension to general anaesthesia and positive pressure ventilation, we found that norepinephrine and dobutamine had the same positive effect on cardiac output, although achieved in distinctly different manners. Dobutamine improved the right ventricular arterio-ventricular coupling, while norepinephrine produced greater left ventricular contractility by improving the placement of the interventricular septum, and therefore increasing the end-diastolic volume of the left ventricle. Further, in the norepinephrine-treated animals, the improvement in cardiac index was accompanied by significantly greater systemic blood pressures.

## Data Availability

The datasets used and/or analysed during the current study are available from the corresponding author on reasonable request.

## Abbreviations

ANOVA: Analysis of variance
BSA: Body surface area
CI: Cardiac index
CO: Cardiac output
CVP: Central venous pressure
DAP: Diastolic arterial pressure
DOB: Dobutamine
d*P*/d*t*_max_: Maximum rate of pressure change
d*P*/d*t*_min_: Minimum rate of pressure change
Ea: Arterial elastance
EDPVR: End-diastolic pressure–volume relationship
ESPVR: End-systolic pressure–volume relationship
ESPVR *V*_o_: End-systolic pressure–volume relationship x-axis intercept
HR: Heart rate
LV: Left ventricle
MAP: Mean arterial pressure
mPAP: Mean pulmonary artery pressure
NE: Norepinephrine
NT-proBNP: N-terminal pro-brain natriuretic peptide
PAB: Pulmonary artery banding
SvO_2_: Mixed venous oxygen saturation
SAP: Systolic arterial pressure
SV: Stroke volume
SVRI: Systemic vascular resistance index
*P*_max_: Maximum ventricular pressure
*P*_min_: Minimum ventricular pressure
PRSW: Preload recruitable stroke work
PVRI: Pulmonary vascular resistance index
RV: Right ventricle
